# Anterior and posterior subareas of the dorsolateral frontal cortex in socially relevant decisions based on masked affect expressions

**DOI:** 10.12688/f1000research.4734.3

**Published:** 2015-07-09

**Authors:** Denise Prochnow, Sascha Brunheim, Hannes Kossack, Simon B. Eickhoff, Hans J. Markowitsch, Rüdiger J. Seitz

**Affiliations:** 1 Department of Neurology, Heinrich-Heine University Düsseldorf, Düsseldorf, D-40225, Germany; 2 Institute for Clinical Neuroscience and Medical Psychology, University of Düsseldorf, Düsseldorf, D-40225, Germany; 3 Department of Psychology, Bielefeld University, Bielefeld, D-33615, Germany

**Keywords:** functional connectivity, dorsolateral frontal cortex, masked affect expressions, functional magnetic resonance imaging, decision-making

## Abstract

Socially-relevant decisions are based on clearly recognizable but also not consciously accessible affective stimuli. We studied the role of the dorsolateral frontal cortex (DLFC) in decision-making on masked affect expressions using functional magnetic resonance imaging. Our paradigm permitted us to capture brain activity during a pre-decision phase when the subjects viewed emotional expressions below the threshold of subjective awareness, and during the decision phase, which was based on verbal descriptions as the choice criterion. Using meta-analytic connectivity modeling, we found that the preparatory phase of the decision was associated with activity in a right-posterior portion of the DLFC featuring co-activations in the left-inferior frontal cortex. During the subsequent decision a right-anterior and more dorsal portion of the DLFC became activated, exhibiting a different co-activation pattern. These results provide evidence for partially independent sub-regions within the DLFC, supporting the notion of dual associative processes in intuitive judgments.

## Introduction

Reading of, and reacting to the numerous dynamic and variable nonverbal signals that are sent out voluntarily or unintentionally in an everyday social situation is challenging and requires the interaction of many brain systems ( Frith & Frith, 2003; Xi *et al.*, 2011). Particularly in social situations, people tend to evaluate their surroundings, including their interaction partner ( Ellsworth & Scherer, 2003). The human face is the most important object for such an evaluation, since it acts as a key component in conveying socially relevant messages in rapid succession ( Ekman & Friesen, 1969). Owing to the complexity of social encounters and the many communicative signals produced by rapidly changing facial expressions, it appears likely that some facial expressions might be too subtle to be perceived fully consciously by the addressee. However, even these transient signals might be of high relevance in “gut-feeling”-based social decisions. For example, inferring even a slightly aggressive emotional state from another’s behavior or facial expression might be crucial for the decision between appeasement in order to avoid confrontation or provocation. Thereby, understanding the mental state of others can be self-profitable for the individual.

The affective primacy hypothesis ( Murphy & Zajonc, 1993) highlights the effects of not consciously perceived affective information, stating that affect can be elicited prior to cognitive processing even when its origin is not consciously accessible. In line with this assumption, studies have shown that subliminal stimuli are processed similarly to consciously accessible stimuli ( Henson *et al.*, 2008; Nomura *et al.*, 2004; Prochnow *et al.*, 2013b). Hence they are able to affect attitudes and judgments which are potent determinants of decision-making in complex situations ( Dimberg *et al.*, 2000; Li *et al.*, 2008; Moskowitz *et al.*, 2012; Ruys & Aarts, 2012; Sweeny *et al.*, 2009; Winkielman *et al.*, 2005).

Decision-making as a term subsumes multiple aspects such as different phases as well as the circumstances of decision-making, such as risky decisions and ambiguous decisions ( Bechara *et al.*, 2005). Typically, gambling paradigms are used to study decision-making ( Bechara *et al.*, 1994; Bechara *et al.*, 2005; Brand *et al.*, 2005; Brand *et al.*, 2006). However, there exist also standardized paradigms with more emphasis on social aspects like the Ultimatum Game or the Prisoner’s Dilemma Game ( Baumgartner *et al.*, 2011; Güth *et al.*, 1982; Sanfey, 2007; van’t Wout *et al.*, 2005). Due to the omnipresence of decisions in everyday life, many different experimental settings are suited to assess socially relevant decisions and decision-making often appears to be implicitly studied in mental state reasoning or theory of mind (ToM) paradigms ( Hall *et al.*, 2010; Hooker *et al.*, 2008; Mériau *et al.*, 2006; Prochnow *et al.*, 2013a; Reniers *et al.*, 2012; Walter *et al.*, 2004). Recent evidence, however, suggests that gambling and ToM scenarios are based at least partly on different neural circuits ( Xi *et al.*, 2011).

Svenson’s “Differentiation and Consolidation Theory” (1996) considers decision-making as the result of a number of different sub-processes. These comprise a pre-decision phase during which different choice alternatives are compared, the decision itself and a post-decision consolidation phase. Following the theory, a number of studies investigated the preparatory processes of different kinds of real-life and gambling decisions and found that the ventromedial frontal cortex (VMFC) and dorsolateral frontal cortex (DLFC) are related to the computation of decision values ( Camus *et al.*, 2009; Hall *et al.*, 2010; Jocham *et al.*, 2012; Litt *et al.*, 2011; Reniers *et al.*, 2012; Sokol-Hessner *et al.*, 2012; van’t Wout *et al.*, 2005). Further evidence suggests that both regions continuously share information during this process ( Baumgartner *et al.*, 2011; Sokol-Hessner *et al.*, 2012), along with other interconnected areas within the prefrontal cortex ( Miller & Cohen, 2001). The DLFC has also been identified as crucially involved in decisions involving ambiguity or uncertainty, paradigms which are considered being predominantly cognitive in nature ( Hosseini *et al.*, 2010; Krain *et al.*, 2006). Accordingly, the DLFC has traditionally been linked to cognitive control and monitoring processes ( Cole & Schneider, 2007; Durston *et al.*, 2003; Milham *et al.*, 2003; Wagner *et al.*, 2001).

However, increasing evidence shows, that DLFC engagement is not limited to decision and judgment tasks in a predominantly cognitive environment but is found in social and affective contexts as well ( Bzdok *et al.*, 2012a; Hall *et al.*, 2010; Lawrence *et al.*, 2006; Opialla *et al.*, 2015; Prochnow *et al.*, 2013a; Prochnow *et al.*, 2013b; Prochnow *et al.*, 2014b; Silvers *et al.*, 2015; Thirioux *et al.*, 2014; Walter *et al.*, 2004). Anatomically, the DLFC has close connections to the parietal and premotor cortices, via the thalamus to the cerebellum ( Hoshi, 2006) and also to regions that have been critically implicated in mentalizing, such as the temporo-parietal junction ( Bzdok *et al.*, 2012b; Kucyi *et al.*, 2012), the anterior cingulate cortex (ACC), and right-inferior frontal gyrus (IFG) ( Cieslik *et al.*, 2013). Notably, in line with previous research highlighting the important role of the DLFC in the preparatory stages of a decision, we found DLFC activity when subjects were presented with either subtle or prominent emotional expressions on which a subsequent decision should be based ( Prochnow *et al.*, 2013b; Prochnow *et al.*, 2014b). Conversely, the DLFC became also engaged late during the actual discrimination and categorization of evolving emotional facial expressions, even when the executive load was partly controlled ( Prochnow *et al.*, 2013a). While in our studies the activation tended to be located in posterior parts of the DLFC during preparation of the decision, it was located more anterior when the decision itself took place.

In the current functional magnetic resonance imaging (fMRI) study we extended the earlier study ( Prochnow *et al.*, 2013b) to investigate the role of the dorsolateral frontal cortex (DLFC) in socially relevant decisions based on subtle emotional information. In the light of our previous results implicating the DLFC both in the preparatory stage of decision-making as well as in the actual decision, our novel paradigm permitted differentiating between both sub-processes within the same decision process. In particular, we presented facial expressions showing very short (40 ms) happy, angry or sad expressions, which were immediately superimposed by a neutral expression of the same actor, which masked the subtle emotional expression the participants had to evaluate. In this preparatory stage of the decision process, the subjects were already aware that a decision had to be made on the basis of the ambiguous facial expression but necessary information to actually make the decision was still lacking. The actual decision could not been made until pairs of emotional adjectives serving as the decision criterion were presented along with the instruction to decide which adjective matched best the previously seen facial expression. This approach permitted us to explore the role of the DLFC in relation to different aspects of socially-relevant decisions.

We hypothesized that the DLFC becomes active when socially relevant decisions based on subtle emotional information which is not accessible to fully conscious perception are made. Specifically, based on our own previous data, as well as evidence from primate studies and network analyses (cf. Hoshi, 2006 for a review; Cieslik *et al.*, 2013), we predicted that the pre-decision phase and subsequent decision engage different subareas within the DLFC, and that this at least partly functional specialization is reflected by different co-activation patterns.

## Materials and methods

The paradigm used in this study and described in detail in the following section has been previously used in another study comparing brain activation patterns between facial expressions of emotion which were either clearly visible or presented below the threshold of subjective awareness ( Prochnow *et al.*, 2013b). It has been designed in order to being able to test different hypotheses related to the processing of subliminal information based on the same paradigm. Other than the previous study, the current work focuses on the decision aspect of the overall paradigm since the subjects were instructed to decide which of two subsequently presented emotional adjectives best described the mood observed in the previously seen face. Furthermore, this paper addresses the novel issue of functional connectivity of the activated lateral prefrontal cortex.

### Participants

The screening of the participants comprised of assessments of handedness (Edinburgh inventory, Oldfield, 1971), alexithymia (TAS-20, Bagby *et al.*, 1994), depressiveness (BDI, Hautzinger *et al.*, 1994), empathy (SPF, German adaptation of the Interpersonal Reactivity Index, http://psydok.sulb.uni-saarland.de/volltexte/2009/2363/pdf/SPF_Artikel.pdf) and affect (PANAS, Watson *et al.*, 1988) in order to only enroll participants with an intact ability to understand emotions and infer emotional states. Exclusion criteria were: left handedness, signs of alexithymia (TAS-20 > 52) or depressiveness (BDI > 9), low self-reported empathy (SPF scale fantasy < 10, SPF scale perspective-taking < 13, SPF scale empathic concern < 12), critical life events during the last year (assessed by means of a short self-developed questionnaire asking whether the participants recently experienced the loss of a beloved one or other traumata), a predominantly negative mood on the day of testing (PANAS negative affect > positive affect), intake of psychotropic drugs or a contraindication of fMRI scanning. Contraindications could be pregnancy, fMRI incompatible or irremovable metals like pacemakers or implants, claustrophobia, and fraction anomalies of sight that could not be corrected by MRI suitable glasses or contact lenses. Participants were recruited using flyers on the university campus. From the 18 participants fulfilling the inclusion criteria for the fMRI study, six were later excluded from data analysis due to movement artifacts or reports of being aware of the subtle emotional expressions indicating a too low threshold of subjective awareness which would have been a confounding factor (see the next section for more information on the debriefing procedure). All participants had normal or corrected-to-normal vision and gave informed written consent to participate in the fMRI study and for publication of the study results. Experiments were approved by the ethics committee of the Heinrich-Heine University Düsseldorf (project # 3614) and conducted according to the Declaration of Helsinki. Statistical data analysis was performed on the data from the remaining 12 healthy volunteers (5 men/7 women) who had a mean age of 23.8 (SD = 3.0) and a median of 16.5 (9–18) years of education.

### Stimulus material and stimulation procedure

During fMRI scanning, participants lay supine in the scanner and viewed the experimental stimuli through a mirror attached to the head coil. The images were presented using presentation software (Version 14.9, Neurobehavioral Systems Inc., Albany CA). During stimulation, participants were presented with male and female facial expressions of emotion depicting happiness, anger or sadness via projection on a semitransparent screen installed in the scanner room using an LCD-projector positioned outside the scanner room (Ekman & Friesen Picture Set, Ekman & Friesen, 1976). Presented were just the faces, while the remaining parts of the heads including the hair and ears were conceiled by the blank of the background. The faces were followed by a blank of 2600 ms on average which was jittered randomly between 400 and 4800 ms. Thereafter, pairs of emotional adjectives were presented as text on screen for 3000 ms (e.g. sorrowful (betrübt) – annoyed (verärgert)) after a jittered (400–4800 ms) time interval. They were instructed to imagine being confronted with someone showing the particular facial expression and to press one of two response buttons (left, right) to decide which adjective corresponded best to the affect of the person depicted. If they felt that none of the adjectives would match, they were requested to choose the best fit (forced choice paradigm).

In 96 experimental trials which were scanned consecutively in one scanning session, the facial expressions of emotion were shown for only 40 ms and then superimposed by a masking neutral expression of the same person for 360 ms. Each emotion (happy, angry, sad) was repeated 32 times in a pseudorandomized order. In addition, there were another 96 trials in which no masking technique was applied and the emotional expression lasted for 400 ms (for a comparison of the masked emotional and unmasked emotional conditions, see Prochnow *et al.*, 2013b). Also, we used scrambled versions of the face images to measure baseline as typically used to map the cerebral areas specifically related to face perception ( Slotnik & White, 2013; Zhu *et al.*, 2013). These scrambled images were produced from the digitized images of the faces used and corrected for luminescence. Thus, the scrambled images had the same visual features as the images of the faces and were presented in an identical manner as the faces. Specifically, the scrambled images of the emotional faces were presented for 400ms in the unmasked condition, while the scrambled images of the emotional faces were presented for 40ms followed by presentation of the scrambled images of the neural faces for 360 ms in the masked condition. Accordingly, the scrambled images were alterations of the original image maintaining the basic visual features but removing meaning from the image.

Masking is a common technique validated by many studies suited to prevent a short stimulus from being consciously perceived (e.g. Dimberg *et al.*, 2000; Suslow *et al.*, 2013). In order to ensure that despite of the masking technique, our subjects were not aware of the masked emotional expression, they were subjected to a post scanning debriefing similar to the one described in Chartrand & Bargh (1996). The debriefing consisted of increasingly precise questions about the assumed goal of the study, the perception of the stimuli and the procedure. Most participants thought the study was about decision-making or subjective judgments of different facial expressions. However, eight participants (26%) had a suspicion that there were emotional faces presented very shortly before the neutral faces. These were excluded from further data analysis. Furthermore, 78% reported to have noticed a flickering in some of the trials, but did not attribute any meaning to this phenomenon. In fact, the flickering could be perceived during the switch between the shortly presented emotional expression and the clearly visible neutral masking expression due to slight details changing in the face as the position of the eyebrows and/or mouth. For comparison, the transition between the facial stimuli and the scrambled image was clearly visible and was thus not perceived as a flickering.

For data analysis, the paradigm outlined above was considered to represent two different time intervals referring to Svenson’s distinct decision steps ( Svenson, 1996). Being presented with the facial stimuli (or the scrambled images in the control condition, respectively) represented the pre-decision phase since the subjects were already aware they were required to make decisions based on the pictures they were presented with. The instruction to choose one out of two adjectives in order to indicate which was a closer match to the facial expression seen before thus prepared subjects for the subsequent decision, in our paradigm the moment they pressed the response button. Accordingly, in the control condition, when only scrambled images were presented, decisions had to be made based on instructions on screen. Thus, beyond brain areas related to visual or face processing and associated mental processes also brain areas related to decision making should become engaged. Visual processing of the word list, the selection of the active finger and the button press were identical in both conditions. 

The “pictures of facial affect” dataset is one of the most intensively studied facial expression datasets of all times (e.g. Adolphs, 2002; Seitz *et al.*, 2008). It contains expressions of six basic emotions, as well as a neutral reference expression of male and female actors. All neutral faces used as masks in the current study were previously rated neutral in a pre-study with 30 volunteers. In the pre-study, the participants were required to rate whether a presented facial expression represented one of the six basic emotions (anger, sadness, fear, disgust, happiness, surprise) or a neutral expression and to which degree (measured in percent) the expression represented each of the emotions or neutrality. In addition, the emotional adjectives used as the response criteria were matched for word frequency, perceived arousal and dominance (SAM, Bradley & Lang, 1994) based on data from another pre-study in 44 volunteers.

### Scanning parameters

Scanning was performed on a 3 T Siemens Trio TIM MRI scanner (Erlangen, Germany) using an EPI-GE sequence (TR = 2000 ms, TE = 30 ms, flip-angle = 90°). The whole brain was covered by 28 transversal slices oriented parallel to the bi-commissural plane (in-plane resolution = 1.5 mm × 1.5 mm, slice thickness = 4.0 mm, interslice gap = 0 mm). In each run, 1200 volumes were acquired. The first three volumes of each session did not enter the analysis. A 3D-T1-weighted image (gradient echo sequence) with high-resolution consisting of 192 sagittal slices and 1 mm × 1 mm resolution was also acquired in each subject (TR = 2300 ms, TE = 2.98 ms, flip angle = 90°).

FMRI scanning was followed by approximately 6 min of anatomical scanning. Post-scanning, participants rated all stimuli on the dimensions arousal, valence and dominance (SAM, Bradley & Lang, 1994) and were debriefed about the experiment.

## Data processing and analysis

### Behavioral data analysis

Behavioral data were analyzed using SPSS software PASW, Predictive Analysis Software, version 20). Prior to analysis, all statistical data were tested for normal distribution using Kolmogorov-Smirnov test. For comparison of means, single factor analyses of variance (ANOVA) were used.

### FMRT data analysis

The Brainvoyager QX software package (Brain Innovation, Maastricht, The Netherlands) was used for the analysis of imaging data. Functional data were pre-processed including Gaussian spatial smoothing (FWHM = 8), temporal filtering, removal of linear trends and movement correction. In each subject, the 2-D slice time-course image data were co-registered with the volumetric 3-D Gradient Echo data sets from the same session.

We analyzed the blood oxygenation level dependent (BOLD) changes in a mixed rapid event-related model and entered the planned contrasts in a random effects group analysis. The whole-brain analysis was based on a general linear model (GLM) and a deconvolution approach which allowed the capturing of event-related brain activity at different time steps after event onset, estimating the hemodynamic response function (HRF). The third volume (4000 ms after event onset) was chosen in order to map activation patterns when the blood oxygen dependent (BOLD) increase was close to peak. The separation of the two defined decision phases was possible by the event-related character of the scanning procedure in which the two events of interest were separated in later data analysis by applying a temporal jitter (temporal separation of pre-decision and decision was 2.600 ms on average) using a scanning repetition time of 2000 ms. In this exploratory study, clusters of activations were considered significant when they surpassed a p < 0.005 and had a minimal cluster size of 405 voxels in 3D space (equivalent to 15 cohesive voxels). This procedure corrects for the limited spatial resolution and the autocorrelation of adjacent voxels in the fMRI images and for multiple comparisons ( Knorr *et al.*, 1993; Worsley *et al.*, 1992). The following regressors were included: *baseline, pre-decision phase, decision phase*, and *motor control*. Scrambled faces (generated by a self-programmed software) served as the baseline condition, and motor control reflected a simple motor response task (reacting towards an unrelated target word out of two words) in order to subtract motor and reading related activity.

In addition to the whole brain analysis, the activated clusters in the DLFC during the preparatory decision phase as well as the decision itself were defined as regions of interest (ROI) in order to extract their parameter estimates (β) for statistical comparison of the degree of activation between conditions. To ensure comparability with data in the open literature, we defined all activated regions within the DLFC as ROIs with a maximum cluster spread range of 10 mm around the peak of activation. All coordinates are given as peak coordinates in stereotactic space ( Talairach & Tournoux, 1988).

### Functional connectivity analyses

We used meta-analytic connectivity modeling (MACM) to explore the task-based functional connectivity of the two ROIs identified in this study in the DLFC. After identification of all experiments in the BrainMap database ( www.brainmap.org; Laird *et al.*, 2011; Laird *et al.*, 2009) which report activation of the seed regions, quantitative meta-analysis permitted testing for convergence across the clusters of activation reflecting co-activation with the seed regions ( Eickhoff *et al.*, 2010). Our analysis was based on approximately 7500 experiments from the BrainMap database reflecting functional mapping studies involving group analyses on healthy participants. Importantly, in order to ensure a completely data-driven approach, all experiments fulfilling the above-mentioned criteria of the BrainMap database were included regardless of behavioral classification. In a first step, all experiments reporting foci within a 5 mm radius of the seed regions were identified ( Cieslik *et al.*, 2011; Eickhoff *et al.*, 2011a), followed by activation likelihood estimation (ALE) to discover co-activations across experiments ( Eickhoff *et al.*, 2010; Eickhoff *et al.*, 2009). Importantly, ALE is based on the assumption that the reported foci are not single points but function as centers for 3D Gaussian probability distributions considering the focus-related spatial uncertainty using an empirical model of between-subject and between-template variance ( Eickhoff *et al.*, 2009). Voxel-wise combination of the probabilities related to all foci then permitted creating modelled activation (MA) maps ( Turkeltaub *et al.*, 2012). These were subsequently merged in order to get voxel-wise and noise-corrected ALE-scores representing the concordance of results at a family-wise error (FWE) corrected p-threshold of p < 0.05 ( Eickhoff *et al.*, 2012).

In a further step, difference maps contrasting functional connectivity maps of the two defined DLFC ROIs were obtained based on their voxel-wise differences as extracted from their MACM-maps. Subsequently, two groups of experiments were formed by pooling and randomly assigning them to same-size groups ( Eickhoff *et al.*, 2011b). A repeated (10,000 times) subtraction of the group’s voxel-wise ALE-scores resulted in an empirical null distribution of ALE-score differences between the two conditions. This was followed by thresholding the map of true differences at a probability of p > 0.95 for a true difference between both. To avoid false positive voxels, the resulting maps were masked with the respective main effect of the minuend connectivity map and the minimal cluster size was 20 cohesive voxels. Thus, in order to show the differences of the connectivity patterns for the two regions in the dorsolateral frontal cortex as obtained from our fMRI-experiment, an empirical null distribution was calcutated by the repeated subtraction of the randomly paired ALE-score maps of the approximately 7500 experiments from the BrainMap database. The noise characteristic of this null distribution map was used subsequently as a reference for thresholding the map of the true ALE-score differences of the two connectivity patterns at p < 0.05.

Statistical data of subareas of the dorsolateral frontal cortex in socially relevant decisions based on masked affect expressions Subject Age EduYears - years of education Sex TAS20 - sum score of TAS-20 BDI - sum score of BDI SPFfantasy - SPF scale fantasy SPFperspect - SPF scale perspective-taking SPFempathy - SPF scale empathy Edingburgh - Handedness CriticalLifeEvents - presence of critical life events PANAS_moment - mood actually PANAS_today - mood today PANAS_generally - mood generally RTimHappy - reaction time subliminal happiness RTimSad - reaction time subliminal sadness RTimAnger - reaction time subliminal anger RTimFace - reaction time all subliminal HITimHappy - hits subliminal happpiness HITimSad - hits subliminal sadness HITimAnger - hits subliminal anger HITimFace - hits subliminal all rpDLFC_PreDec - posterior dorsolateral frontal cortex pre-decision rpDLFC_Dec - posterior dorsolateral frontal cortex decision raDLFC_PreDec - anterior dorsolateral frontal cortex pre-decision raDLFC_Dec - anterior dorsolateral frontal cortex decision

## Results

The fMRI study was preceded by a behavioral study in 32 healthy subjects (mean age 23.9 years, SD = 2.3) testing whether the experimental manipulation was successful (cf. Prochnow *et al.*, 2013b). We found that the subtle masked facial expressions of emotion affected the adjective choice and were thus suitable for a study on decision-making (for a detailed description of the statistical results, please refer to Prochnow *et al.*, 2013b).

We first present the activation patterns obtained by whole-brain analysis with emphasis on the masked facial expressions of emotion at the pre-decision phase and the subsequent actual decision. Second, we report the comparisons based on the regionally extracted parameter estimates (β) for the two activated areas in DLFC. And finally, we describe the functional connectivity of these seed regions in DLFC.

### Activation patterns in whole brain analysis

**Figure 1.**
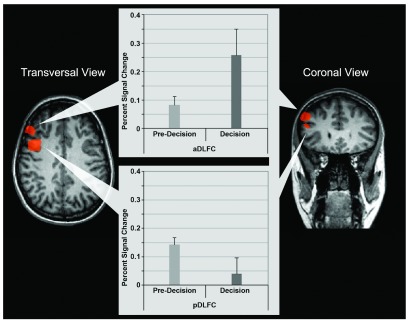
Location of the activated brain regions in DLFC that became activated in the pre-decision phase (pDLFC) and during the subsequent decision (aDLFC). These activation clusters were used to define regions of interest based on their activation peaks plus a cluster spread range of 10 mm. The diagrams show their degrees in percent signal change at both events of interest.

***Pre-decision phase: masked facial expressions vs. baseline.*** In the pre-decision phase, comparing masked emotional facial expressions with scrambled images of faces (baseline) resulted in a bilateral activation of the occipital cortex extending to the fusiform gyrus, of the caudal intraparietal sulcus, as well as of the right superior temporal sulcus, left premotor cortex and most importantly of a right posterior portion of the DLFC ( *x* = *44*, *y* = *16*, *z* = *27*, Figure 1).

***Decision phase: decisions based on masked affect expressions vs. motor control.*** At the moment of the actual decision as indicated by the subjects choice of one of two emotional adjectives following a masked emotional face, we found activation of the left cuneus, left putamen, left paracingulate gyrus, right inferior frontal gyrus and, most importantly, of an anterior portion of the right DLFC ( *x* = *50*, *y* = *28*, *z* = *36*, Figure 1).

***Region of interest (ROI) analysis.*** The activation peak of the ROI related to *pre-decisional masked face presentation* was located posterior within the DLFC, while the activation peak of the ROI related to the *decision phase* was located more anterior with a Euclidean distance of 16.16 mm to the ROI related to *pre-decisional masked face presentation.* This distance exceeded the spatial resolution of the fMRI images (8 mm full width and half maximum (FWHM)).

We conducted pairwise t-tests to compare parameter estimates between the two DLFC ROIs (for their definition see the Materials and methods section) at α = 0.05, and additionally calculated effect sizes (Cohen’s d) due to the small sample size. The parameter estimates related to *pre-decisional masked face presentation* did not differ significantly from those during the *decision phase* (T = -1.02, df = 11, p = 0.329; Cohen’s d = 0.2).

Correlation analyses revealed that no correlation was found between parameter estimates related to *pre-decisional masked face presentation* and the *decision phase*. Notably, the parameter estimates of the *decision phase of the masked emotional faces* correlated significantly with the accuracy of related decisions following sad expressions. However, parameter estimates in none of the defined DLFC ROIs correlated with self-reported empathy (SPF questionnaire), mood (BDI, Hautzinger *et al.*, 1994) or emotional competence (TAS-20, Bagby *et al.*, 1994).

***Functional connectivity analyses.*** This hypothesis-free meta-analytic approach capitalized on the large BrainMap database for calculating statistical maps of co-activation for these two seed regions. For their computation using ALE-based meta-analysis, the posterior ROI related to *pre-decisional masked facial expressions* and the anterior ROI related to the actual *decision phase* in the DLFC were used as seed regions. Both were associated with bilateral co-activations in the DLFC and the adjacent premotor cortex. Also, there was task-dependent co-activation in the dorsomedial frontal cortex and around the intraparietal sulcus which was found bilaterally in relation to the seed region associated with *pre-decisional masked facial expressions* and exclusively right-sided regarding the seed region representing the subsequent *decision phase*. In addition, the seed region in the DLFC related to *pre-decisional masked facial expressions* featured co-activations in the inferior frontal gyrus bilaterally and in the left fusiform gyrus.

**Figure 2.**
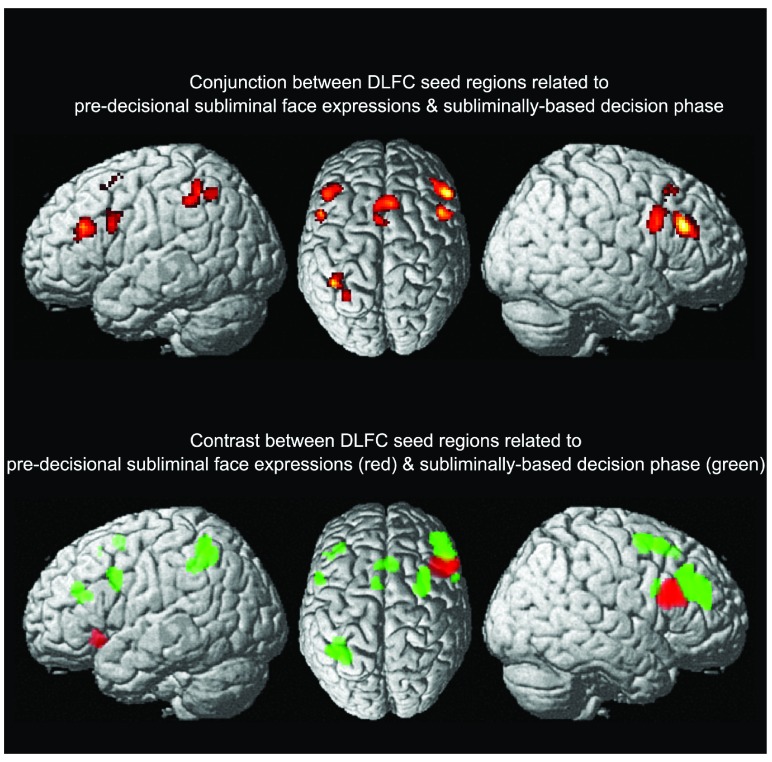
Co-activation maps of the conjunction of co-activations related to the two DLFC seed regions (top), and the difference maps related to the pre-decisional masked facial expressions (bottom red) and the subsequent related decision phase (bottom green).

The conjunction between co-activations related to both DLFC seed regions comprised two clusters of co-activations in the DLFC, one located more anterior and the other more posterior, a cluster in the left intraparietal sulcus and a cluster in the dorsomedial frontal cortex which included parts of the pre-supplementary motor area (pre-SMA) ( Figure 2).

Contrasting the co-activation patterns between the two seed regions yielded a more distributed pattern of co-activated clusters in relation to the DLFC seed region associated with the *decision phase*. This seed region featured stronger co-activations in the left and right DLFC, the adjacent premotor cortex, the dorsomedial frontal cortex, the left pre-SMA and around the left intraparietal sulcus ( Figure 2). Interestingly, the seed region in relation to the *decision phase* was associated with stronger co-activations in two distinct DLFC clusters bilaterally, an anterior and a posterior one, whereas the seed region of *pre-decisional masked facial expressions* featured a stronger co-activation in a right DLFC region located between these two clusters. Also, it was associated with stronger co-activations in the right inferior frontal gyrus ( Figure 2).

## Discussion

This study aimed at identifying the brain areas related to different aspects of decision-making based on masked emotional information that presented a model of daily interpersonal interactions. Specifically, we used a paradigm capable of distinguishing the activation patterns during a preparatory decision phase when not all decision-relevant information was present, from activation patterns related to the decision itself. We found the right DLFC to be involved in both decision stages at clearly different positions: a posterior portion became activated when the actual decision was made as indicated by the subject’s button press ( *decision*). The *pre-decision phase* during which the subjects were presented with masked emotional facial expressions, which they had to evaluate later, was associated with an activation increase in the right anterior DLFC. No significant differences were found in the degree of activation between both sub-regions, as indicated by the extracted parameter estimates.

There is a large body of evidence implicating the DLFC in decision-making tasks ( Basten *et al.*, 2010; Domenech & Dreher, 2010; Gilbert *et al.*, 2010; Hall *et al.*, 2010; Hayama & Rugg, 2009; Hosseini *et al.*, 2010; Huettel & Misiurek, 2004; Plassmann *et al.*, 2007), especially when the decisions are characterized by some degree of ambiguity ( Christakou *et al.*, 2009; Kahnt *et al.*, 2011; Krain *et al.*, 2006). Moreover, DLFC activity has been found in various higher-order cognitive tasks such as working memory and monitoring tasks ( Durston *et al.*, 2003; Kellermann *et al.*, 2012; Opitz *et al.*, 2000; Wagner *et al.*, 2001) and cognitive control tasks ( Cieslik *et al.*, 2010; Cole & Schneider, 2007; Eickhoff & Grefkes, 2011; Jakobs *et al.*, 2009; Milham *et al.*, 2003). These are considered pre-dominantly “cold” cognitively-driven tasks ( Zelazo & Muller, 2002) and may act as key players in self-related control tasks such as decision-making and choice (reviewed by Banfield *et al.*, 2004).

However, even though affect-based decisions have been traditionally linked to the recruitment of the ventromedial and orbitofrontal prefrontal cortex, which we failed to observe in the current study ( Chib *et al.*, 2009; Grabenhorst & Rolls, 2011; Krain *et al.*, 2006; Smith *et al.*, 2010; Zelazo & Muller, 2002), we consistently found DLFC activation in affective judgment tasks ( Prochnow *et al.*, 2013a; Prochnow *et al.*, 2013b; Prochnow *et al.*, 2014b). Our observations are supported by studies using affective tasks which implicitly studied decisions in an affective context ( Bzdok *et al.*, 2012a; Lawrence *et al.*, 2006; Opialla *et al.*, 2015; Silvers *et al.*, 2015; Thirioux *et al.*, 2014; Walter *et al.*, 2004). In order to model daily interpersonal interactions we intentionally created a decision-making paradigm in which the subjects had to base their decisions on subtle and thus ambiguous facial expressions. Following the affective primacy hypothesis ( Murphy & Zajonc, 1993), the emotional expressions were considered to elicit an affective response in the observer even though the subjects were not aware of having seen them, similarly as to what Ekman has described as micro expressions ( Ekman, 1992; Shen *et al.*, 2012). The short emotional expression was thus expected to add an emotional flavor onto the masking neutral expression which loaded an ambiguous stimulus with a specific emotional state ( Rohr *et al.*, 2012; Prochnow *et al.*, 2013b).

In the current study, as well as in previous studies ( Prochnow *et al.*, 2013b; Prochnow *et al.*, 2014b), we show that already during the presentation of *pre-decisional masked facial expressions* a posterior and more ventral portion of the DLFC became activated. According to anatomical coordinates, this activation cluster corresponded to dorsolateral frontal regions found in normative decision-making ( Baumgartner *et al.*, 2011) and ill-structured problem-solving ( Gilbert *et al.*, 2010), indicating its importance in the decision-making process. During this preparatory stage of decision-making, when not all necessary information to make a goal-directed decision is present, Svenson’s theory assumes that calculation of decision values takes place ( Svenson, 1996). Evidence for the involvement of the DLFC in the calculation of decision values comes from a growing number of studies ( Camus *et al.*, 2009; Litt *et al.*, 2011; Plassmann *et al.*, 2007; Sokol-Hessner *et al.*, 2012). Notably, a more anterior and dorsal portion of the DLFC became activated when the adjectives offered as the decision criteria were presented and the subjects had to make a decision (forced choice paradigm). This result is in line with our previous study showing anterior DLFC engagement during online emotion discrimination and categorization ( Prochnow *et al.*, 2013a) and suggests that the anterior portion of the DLFC is associated with uncertain decisions ( Hosseini *et al.*, 2010).

DLFC activations reported in the literature are heterogeneous in their locations and also regarding their related tasks. Most clusters are situated in close proximity to the anterior cluster found here or even more anterior. Functionally, they are referred to working memory and monitoring ( Rottschy *et al.*, 2012; Wagner *et al.*, 2001), self-reflection ( Herwig *et al.*, 2012), cognitive control or cognitive conflict ( Cieslik *et al.*, 2010; Eickhoff & Grefkes, 2011; Jakobs *et al.*, 2009; Milham *et al.*, 2003) and different aspects of decision-making ( Krain *et al.*, 2006; Plassmann *et al.*, 2007; Prochnow *et al.*, 2013a). Especially, there seems to be a conceptual overlap of studies examining cognitive control, cognitive conflict and decision-making depending on the focus of the study. Whereas studies focusing on decision-making, including the current study, implicitly study aspects of cognitive control, studies on cognitive control appear to imply aspects of decision-making. In order to get further insights into the functional connectivity of the DLFC, this study also focused on the identification of co-activations of the two subareas within the DLFC obtained in the whole brain analysis.

The analyses of functional connectivity showed that the posterior DLFC cluster activated during the *pre-decision phase* featured stronger co-activations in the right inferior frontal gyrus (IFG) and in a DLFC area located between the precentral and inferior frontal sulcus. By contrast, the anterior portion of the DLFC that became activated during the *actual decision* was associated with stronger co-activations in two DLFC areas framing the DLFC region co-activated in relation to the posterior DLFC seed region. In addition, it featured co-activations of the premotor cortex, a dorsomedial frontal region, the left pre-SMA and the left intraparietal sulcus. Activation of the IFG has been found repeatedly in tasks involving low-level empathy ( Carr *et al.*, 2003; Lamm *et al.*, 2007; Lindenberg *et al.*, 2012; Prochnow *et al.*, 2013a; Schulte-Rüther *et al.*, 2007; Seitz *et al.*, 2008; Shamay-Tsoory *et al.*, 2009), most likely because it is considered an important node of the putative human mirror neuron system ( Rizzolatti & Craighero, 2004). Moreover, the left IFG is well known to accommodate Broca’s speech area ( Lindenberg *et al.*, 2007) and its activation might therefore also reflect covert speech. Accordingly, in our paradigm one would expect left IFG activity to co-occur during the *actual decision* since at this stage, the subjects were confronted with verbal descriptions in form of two emotional adjectives they were required to choose in order to respond. Instead, the whole brain analysis showed an activation increase in the right inferior frontal gyrus during the *actual decision*, and neither the *pre-decision phase*, nor the *actual decision* was associated with an activation increase in the left IFG in this sample. However, although the *pre-decision phase* does not involve any explicit speech component, it remains impossible to control for covert speech in fMRI tasks like ours.

Interestingly, in the current study activity in the anterior portion of the DLFC associated with the *actual decision* was also accompanied by an activation increase in the left paracingulate gyrus. This dorsomedial prefrontal region has been found relevant for rapid interpersonal evaluations ( Cooper *et al.*, 2012) and theory of mind ( Hooker *et al.*, 2008; Schulte-Rüther *et al.*, 2007). Moreover, the adjacent pre-SMA has been shown to be crucial in the context of the generation of the so-called Bereitschaftspotential to perform a movement ( Shibasaki & Hallett, 2006), as well as for movement selection ( Deiber *et al.*, 1991; Hoffstaedter *et al.*, 2013). Interestingly, it was not only found active during the recognition of emotions in static emotional facial expressions ( Seitz *et al.*, 2008) but also when dynamically evolving emotional facial expressions had to be discriminated ( Prochnow *et al.*, 2013a). These observations suggest that the dorsomedial portion of the prefrontal cortex including the adjacent pre-SMA becomes involved when an external mental state needs to be transferred into an internal frame of reference ( Seitz *et al.*, 2006; Seitz *et al.*, 2009).

In addition to the identification of different patterns of functional connectivity between the posterior DLFC region related to the *pre-decision phase* and the anterior region related to the *decision phase*, we were interested in the co-activations shared by both DLFC regions. These were bilateral anterior and posterior areas in the DLFC, the dorsomedial frontal cortex including the pre-SMA and the left intraparietal sulcus, suggesting a common network allowing for visuo-spatial and time-related attention ( Culham & Kanwisher, 2001; Davranche *et al.*, 2011; Grefkes & Fink, 2005) and self-referential valuation ( Seitz *et al.*, 2006; Seitz *et al.*, 2009).

In the current study, activations of the two subregions in the DLFC were clearly lateralized to the right cerebral hemisphere featuring co-activations distributed over both hemispheres. This result corresponds to behavioral evidence showing that not consciously accessible faces affected choices regardless of the visual hemifield to which they were presented while, in contrast, subliminally presented words affected choices only when they were presented to the left cerebral hemisphere ( Henke *et al.*, 1994).

Possible limitations of the current study should not go unmentioned. We considered the moment when our subjects viewed the emotional masked facial expressions the preparatory stage of the actual decision since not all relevant information was present to make a goal-directed choice. It cannot, however, be ruled out that instead of measuring a pre-decision phase and the actual decision, there were two different decisions following one-another. A first partial decision based on only the visual information and the outside of subjective awareness elicited affective response and a subsequent decision when the emotional adjectives as the decision criterion were available. For example, Wunderlich *et al.* (2010) provided evidence that people are able to partially make a choice in stimulus space before knowing the motor mapping associated with the final decision. Independent of these theoretical considerations, our fMRI and functional connectivity data showed that both time points were associated with the involvement of different parts of the DLFC indicating functional specialization in the DLFC. Instead of representing a pre-decision phase and the decision itself, the anterior-posterior subdivision could also reflect different degrees to which the decision was goal-directed. Moreover, we used scrambled versions of the digitzed images of the faces as baseline that had been corrected for luminescence, as they were as much as possible identical to the faces except for their appearance. They were scrambled images of the emotional faces in the unmasked condition and scrambled images of the emotional faces followed by scrambled images of the neutral faces in the masked condition. Notably, neutral faces were not appropriate for baseline, since we used images of faces that had been classified as neutral ( Prochnow *et al.*, 2013b) for masking of the emotional face expressions. Moreover, distorted faces or face-like objects would introduce visual features that would attract the subjects‘ attention in a strong and poorly controlled fashion, since they are strong visual stimuli ( Dalrymple *et al.*, 2014; Freeman *et al.*, 2010). Thus, we believe that in the pre-decision phase our paradigm was focussed on the processing of emotional face expressions. Finally, in our event-related protocol we sought to separate the hemodynamic response related to the emotional face expressions in the pre-decision phase from the hemodynamic response related to the verbal stimuli in the decision phase in one experimental paradigm. For this purpose these two visual stimuli were separated by a blank of 2600 ms on average which was jittered between 400 and 4800 ms. Given a TR of 2000 ms this interval was sufficiently long to detect the two different hemodynamic responses, since event-related fMRI can resolve subsequent hemodynamic responses with a separation of as little as 200 to 400 ms ( de Zwart *et al.*, 2009).

## Conclusions

In conclusion, our data suggest that the DLFC is crucial for decisions involving masked, and thus, ambiguous affective information. Moreover, by use of categorical and functional connectivity image analysis approaches we provide evidence for partially independent sub-regions within the right DLFC. Whereas the posterior portion of the right DLFC was relevant for the preparatory phase within the decision process when not all the necessary information for a goal-directed choice were available, the anterior sub-region appeared to be related to later goal-directed decision stages involving sustained attention for time, space and valuation. These results may be related to the notion of dual associative processes in intuitive judgments ( Morewedge & Kahneman, 2010).

## Participant consent

All participants gave informed written consent to participate in the fMRI study. Experiments were approved by the local ethics committee and conducted according to the Declaration of Helsinki.

## Data availability

*figshare:* Statistical data of subareas of the dorsolateral frontal cortex in socially relevant decisions based on masked affect expressions. Doi: 10.6084/m9.figshare.1153792 ( Prochnow *et al.*, 2014a).

## References

[ref-1] Adolphs R: Neural systems for recognizing emotion. *Curr Opin Neurobiol.* 2002;12(2):169–177. 10.1016/S0959-4388(02)00301-X 12015233

[ref-2] Bagby RM Parker JD Taylor GJ: The twenty-item Toronto Alexithymia Scale--I. Item selection and cross-validation of the factor structure. *J Psychosom Res.* 1994;38(1):23–32. 10.1016/0022-3999(94)90005-1 8126686

[ref-3] Banfield JF Wyland CL Macrae CN: The cognitive neuroscience of self-regulation. In Baumeister, R.F. & Vohs, K.D. (Eds.) *Handbook of self-regulation: Research, theory, and applications* New York: Guilford Press,2004;62–83. Reference Source

[ref-4] Basten U Biele G Heekeren HR: How the brain integrates costs and benefits during decision making. *Proc Natl Acad Sci U S A.* 2010;107(50):21767–21772. 10.1073/pnas.0908104107 21118983 PMC3003102

[ref-5] Baumgartner T Knoch D Hotz P: Dorsolateral and ventromedial prefrontal cortex orchestrate normative choice. *Nat Neurosci.* 2011;14(11):1468–1474. 10.1038/nn.2933 21964488

[ref-6] Bechara A Damasio AR Damasio H: Insensitivity to future consequences following damage to human prefrontal cortex. *Cognition.* 1994;50(1–3):7–15. 10.1016/0010-0277(94)90018-3 8039375

[ref-7] Bechara A Damasio H Tranel D: The Iowa Gambling Task and the somatic marker hypothesis: some questions and answers. *Trends Cogn Sci.* 2005;9(4):159–162. 10.1016/j.tics.2005.02.002 15808493

[ref-8] Bradley MM Lang PJ: Measuring emotion: the Self-Assessment Manikin and the Semantic Differential. *J Behav Ther Exp Psychiatry.* 1994;25(1):49–59. 10.1016/0005-7916(94)90063-9 7962581

[ref-9] Brand M Fujiwara E Borsutzky S: Decision-making deficits of korsakoff patients in a new gambling task with explicit rules: associations with executive functions. *Neuropsychology.* 2005;19(3):267–277. 10.1037/0894-4105.19.3.267 15910113

[ref-10] Brand M Labudda K Markowitsch HJ: Neuropsychological correlates of decision-making in ambiguous and risky situations. *Neural Netw.* 2006;19(8):1266–1276. 10.1016/j.neunet.2006.03.001 16942857

[ref-11] Bzdok D Langner R Hoffstaedter F: The modular neuroarchitecture of social judgments on faces. *Cereb Cortex.* 2012a;22(4):951–961. 10.1093/cercor/bhr166 21725038 PMC3450920

[ref-12] Bzdok D Schilbach L Vogeley K: Parsing the neural correlates of moral cognition: ALE meta-analysis on morality, theory of mind, and empathy. *Brain Struct Funct.* 2012b;217(4):783–796. 10.1007/s00429-012-0380-y 22270812 PMC3445793

[ref-13] Camus M Halelamien N Plassmann H: Repetitive transcranial magnetic stimulation over the right dorsolateral prefrontal cortex decreases valuations during food choices. *Eur J Neurosci.* 2009;30(10):1980–1988. 10.1111/j.1460-9568.2009.06991.x 19912330

[ref-14] Carr L Iacoboni M Dubeau MC: Neural mechanisms of empathy in humans: a relay from neural systems for imitation to limbic areas. *Proc Natl Acad Sci U S A.* 2003;100(9):5497–5502. 10.1073/pnas.0935845100 12682281 PMC154373

[ref-15] Chartrand TL Bargh JA: Automatic activation of impression formation and memorization goals: Nonconscious goal priming reproduces effects of explicit task instructions. *J Pers Soc Psychol.* 1996;71(3):464–478. 10.1037/0022-3514.71.3.464

[ref-16] Chib VS Rangel A Shimojo S: Evidence for a common representation of decision values for dissimilar goods in human ventromedial prefrontal cortex. *J Neurosci.* 2009;29(39):12315–12320. 10.1523/JNEUROSCI.2575-09.2009 19793990 PMC6666137

[ref-17] Christakou A Brammer M Giampietro V: Right ventromedial and dorsolateral prefrontal cortices mediate adaptive decisions under ambiguity by integrating choice utility and outcome evaluation. *J Neurosci.* 2009;29(35):11020–11028. 10.1523/JNEUROSCI.1279-09.2009 19726660 PMC6665528

[ref-18] Cieslik EC Zilles K Caspers S: Is there “one” DLPFC in cognitive action control? Evidence for heterogeneity from co-activation-based parcellation. *Cereb Cortex.* 2013;23(11):2677–89. 10.1093/cercor/bhs256 22918987 PMC3792742

[ref-19] Cieslik EC Zilles K Grefkes C: Dynamic interactions in the fronto-parietal network during a manual stimulus-response compatibility task. *Neuroimage.* 2011;58(3):860–869. 10.1016/j.neuroimage.2011.05.089 21708271 PMC7998039

[ref-20] Cieslik EC Zilles K Kurth F: Dissociating bottom-up and top-down processes in a manual stimulus-response compatibility task. *J Neurophysiol.* 2010;104(3):1472–1483. 10.1152/jn.00261.2010 20573974 PMC2944686

[ref-21] Cole MW Schneider W: The cognitive control network: Integrated cortical regions with dissociable functions. *Neuroimage.* 2007;37(1):343–360. 10.1016/j.neuroimage.2007.03.071 17553704

[ref-22] Cooper JC Dunne S Furey T: Dorsomedial prefrontal cortex mediates rapid evaluations predicting the outcome of romantic interactions. *J Neurosci.* 2012;32(45):15647–15656. 10.1523/JNEUROSCI.2558-12.2012 23136406 PMC3513285

[ref-23] Culham JC Kanwisher NG: Neuroimaging of cognitive functions in human parietal cortex. *Curr Opin Neurobiol.* 2001;11(2):157–163. 10.1016/S0959-4388(00)00191-4 11301234

[ref-24] Dalrymple KA Davies-Thompson J Oruc I: Spontaneous perceptual facial distortions correlate with ventral occipitotemporal activity. *Neuropsychologia.* 2014;59:179–191. 10.1016/j.neuropsychologia.2014.05.005 24859691

[ref-25] Davranche K Nazarian B Vidal F: Orienting attention in time activates left intraparietal sulcus for both perceptual and motor task goals. *J Cogn Neurosci.* 2011;23(11):3318–3330. 10.1162/jocn_a_00030 21452942

[ref-26] Deiber MP Passingham RE Colebatch JG: Cortical areas and the selection of movement: a study with positron emission tomography. *Exp Brain Res.* 1991;84(2):393–402. 10.1007/BF00231461 2065746

[ref-27] De Zwart JA van Gelderen P Jansma JM: Hemodynamic nonlinearities affect BOLD fMRI response timing and amplitude. *Neuroimage.* 2009;47(4):1649–1658. 10.1016/j.neuroimage.2009.06.001 19520175 PMC2731556

[ref-28] Dimberg U Thunberg M Elmehed K: Unconscious facial reactions to emotional facial expressions. *Psychol Sci.* 2000;11(1):86–89. 10.1111/1467-9280.00221 11228851

[ref-29] Domenech P Dreher JC: Decision threshold modulation in the human brain. *J Neurosci.* 2010;30(43):14305–14317. 10.1523/JNEUROSCI.2371-10.2010 20980586 PMC6634811

[ref-30] Durston S Davidson MC Thomas KM: Parametric manipulation of conflict and response competition using rapid mixed-trial event-related fMRI. *Neuroimage.* 2003;20(4):2135–2141. 10.1016/j.neuroimage.2003.08.004 14683717

[ref-31] Eickhoff SB Bzdok D Laird AR: Activation likelihood estimation meta-analysis revisited. *Neuroimage.* 2012;59(3):2349–2361. 10.1016/j.neuroimage.2011.09.017 21963913 PMC3254820

[ref-32] Eickhoff SB Bzdok D Laird AR: Co-activation patterns distinguish cortical modules, their connectivity and functional differentiation. *Neuroimage.* 2011b;57(3):938–949. 10.1016/j.neuroimage.2011.05.021 21609770 PMC3129435

[ref-33] Eickhoff SB Grefkes C: Approaches for the integrated analysis of structure, function and connectivity of the human brain. *Clin EEG Neurosci.* 2011;42(2):107–121. 10.1177/155005941104200211 21675600 PMC8005855

[ref-34] Eickhoff SB Jbabdi S Caspers S: Anatomical and functional connectivity of cytoarchitectonic areas within the human parietal operculum. *J Neurosci.* 2010;30(18):6409–6421. 10.1523/JNEUROSCI.5664-09.2010 20445067 PMC4791040

[ref-35] Eickhoff SB Laird AR Grefkes C: Coordinate-based activation likelihood estimation meta-analysis of neuroimaging data: a random-effects approach based on empirical estimates of spatial uncertainty. *Hum Brain Mapp.* 2009;30(9):2907–2926. 10.1002/hbm.20718 19172646 PMC2872071

[ref-36] Eickhoff SB Pomjanski W Jakobs O: Neural correlates of developing and adapting behavioral biases in speeded choice reactions--an fMRI study on predictive motor coding. *Cereb Cortex.* 2011a;21(5):1178–1191. 10.1093/cercor/bhq188 20956614

[ref-37] Ekman P: Facial expressions of emotion: an old controversy and new findings. *Philos Trans R Soc Lond B Biol Sci.* 1992;335(1273):63–69. 10.1098/rstb.1992.0008 1348139

[ref-38] Ekman P Friesen WV: Nonverbal leakage and clues to deception. *Psychiatry.* 1969;32(1):88–106. 5779090 10.1080/00332747.1969.11023575

[ref-39] Ekman P Friesen WV: Pictures of facial affect. Palo Alto, CA Consulting Psychologists Press.1976 Reference Source

[ref-40] Ellsworth PC Scherer KR: Appraisal processes in emotion. In R. J. Davidson, K. R. Scherer, & H. Goldsmith (Eds.), *Handbook of Affective Sciences* Oxford, UK: Oxford University Press.2003;572–595. Reference Source

[ref-41] Freeman JB Rule NO Adams RB Jr: The neural basis of categorical face perception: graded representations of face gender in fusiform and orbitofrontal cortices. *Cereb Cortex.* 2010;20(6):1314–22. 10.1093/cercor/bhp195 19767310

[ref-42] Frith U Frith CD: Development and neurophysiology of mentalizing. *Philos Trans R Soc Lond B Biol Sci.* 2003;358(1431):459–473. 10.1098/rstb.2002.1218 12689373 PMC1693139

[ref-43] Gilbert SJ Zamenopoulos T Alexiou K: Involvement of right dorsolateral prefrontal cortex in ill-structured design cognition: an fMRI study. *Brain Res.* 2010;1312:79–88. 10.1016/j.brainres.2009.11.045 19948156

[ref-44] Grabenhorst F Rolls ET: Value, pleasure and choice in the ventral prefrontal cortex. *Trends Cogn Sci.* 2011;15(2):56–67. 10.1016/j.tics.2010.12.004 21216655

[ref-45] Grefkes C Fink GR: The functional organization of the intraparietal sulcus in humans and monkeys. *J Anat.* 2005;207(1):3–17. 10.1111/j.1469-7580.2005.00426.x 16011542 PMC1571496

[ref-46] Güth W Schmittberger R Schwarze B: An experimental analysis of ultimatum bargaining. *J Econ Behav Organ.* 1982;3(4):367–388. 10.1016/0167-2681(82)90011-7

[ref-47] Hall J Whalley HC McKirdy JW: A common neural system mediating two different forms of social judgement. *Psychol Med.* 2010;40(7):1183–1192. 10.1017/S0033291709991395 19811702

[ref-48] Hautzinger M Bailer M Worall H: Beck Depressions-Inventar (BDI). Testhandbuch, Huber, Bern.1994 Reference Source

[ref-49] Hayama HR Rugg MD: Right dorsolateral prefrontal cortex is engaged during post-retrieval processing of both episodic and semantic information. *Neuropsychologia.* 2009;47(12):2409–2416. 10.1016/j.neuropsychologia.2009.04.010 19383503 PMC2712584

[ref-50] Henke K Landis T Markowitsch HJ: Subliminal perception of words and faces. *Int J Neurosci.* 1994;75(3–4):181–187. 10.3109/00207459408986302 8050860

[ref-51] Henson RN Mouchlianitis E Matthews WJ: Electrophysiological correlates of masked face priming. *Neuroimage.* 2008;40(2):884–895. 10.1016/j.neuroimage.2007.12.003 18234522 PMC2516482

[ref-52] Herwig U Kaffenberger T Schell C: Neural activity associated with self-reflection. *BMC Neurosci.* 2012;13:52. 10.1186/1471-2202-13-52 22624857 PMC3483694

[ref-53] Hoffstaedter F Grefkes C Zilles K: The “what” and “when” of self-Initiated movements. *Cereb Cortex.* 2013;23(3):520–30. 10.1093/cercor/bhr391 22414772 PMC3593700

[ref-54] Hooker CI Verosky SC Germine LT: Mentalizing about emotion and its relationship to empathy. *Soc Cogn Affect Neurosci.* 2008;3(3):204–217. 10.1093/scan/nsn019 19015112 PMC2566770

[ref-55] Hoshi E: Functional specialization within the dorsolateral prefrontal cortex: a review of anatomical and physiological studies of non-human primates. *Neurosci Res.* 2006;54(2):73–84. 10.1016/j.neures.2005.10.013 16310877

[ref-56] Hosseini SM Rostami M Yomogida Y: Aging and decision making under uncertainty: behavioral and neural evidence for the preservation of decision making in the absence of learning in old age. *Neuroimage.* 2010;52(4):1514–1520. 10.1016/j.neuroimage.2010.05.008 20472072

[ref-57] Huettel SA Misiurek J: Modulation of prefrontal cortex activity by information toward a decision rule. *Neuroreport.* 2004;15(12):18883–1886. 15305129 10.1097/00001756-200408260-00009

[ref-58] Jakobs O Wang LE Dafotakis M: Effects of timing and movement uncertainty implicate the temporo-parietal junction in the prediction of forthcoming motor actions. *Neuroimage.* 2009;47(2):667–677. 10.1016/j.neuroimage.2009.04.065 19398017 PMC8019092

[ref-59] Jocham G Hunt LT Near J: A mechanism for value-guided choice based on the excitation-inhibition balance in prefrontal cortex. *Nat Neurosci.* 2012;15(7):960–961. 10.1038/nn.3140 22706268 PMC4050076

[ref-60] Kahnt T Heinzle J Park SQ: Decoding different roles for vmPFC and dlPFC in multi-attribute decision making. *Neuroimage.* 2011;56(2):709–715. 10.1016/j.neuroimage.2010.05.058 20510371

[ref-61] Kellermann TS Sternkopf MA Schneider F: Modulating the processing of emotional stimuli by cognitive demand. *Soc Cogn Affect Neurosci.* 2012;7(3):263–273. 10.1093/scan/nsq104 21258093 PMC3304476

[ref-62] Knorr U Weder B Kleinschmidt A: Identification of task-specific rCBF changes in individual subjects: validation and application for PET. *J Comput Assist Tomogr.* 1993;17(4):517–528. 8331220 10.1097/00004728-199307000-00002

[ref-63] Krain AL Wilson AM Arbuckle R: Distinct neural mechanisms of risk and ambiguity: a meta-analysis of decision-making. *Neuroimage.* 2006;32(1):477–484. 10.1016/j.neuroimage.2006.02.047 16632383

[ref-64] Kucyi A Hodaie M Davis KD: Lateralization in intrinsic functional connectivity of the temporoparietal junction with salience- and attention-related brain networks. *J Neurophysiol.* 2012;108(12):3382–3392. 10.1152/jn.00674.2012 23019004

[ref-65] Laird AR Eickhoff SB Fox PM: The BrainMap strategy for standardization, sharing, and meta-analysis of neuroimaging data. *BMC Res Notes.* 2011;4:349. 10.1186/1756-0500-4-349 21906305 PMC3180707

[ref-66] Laird AR Eickhoff SB Kurth F: ALE Meta-Analysis Workflows Via the Brainmap Database: Progress Towards A Probabilistic Functional Brain Atlas. *Front Neuroinform.* 2009;3:23. 10.3389/neuro.11.023.2009 19636392 PMC2715269

[ref-67] Lamm C Batson CD Decety J: The neural substrate of human empathy: effects of perspective-taking and cognitive appraisal. *J Cogn Neurosci.* 2007;19(1):42–58. 10.1162/jocn.2007.19.1.42 17214562

[ref-68] Lawrence EJ Shaw P Giampietro VP: The role of ‘shared representations’ in social perception and empathy: an fMRI study. *Neuroimage.* 2006;29(4):1173–1184. 10.1016/j.neuroimage.2005.09.001 16337816

[ref-69] Li W Zinbarg RE Boehm SG: Neural and behavioral evidence for affective priming from unconsciously perceived emotional facial expressions and the influence of trait anxiety. *J Cogn Neurosci.* 2008;20(1):95–107. 10.1162/jocn.2008.20006 17919076

[ref-70] Lindenberg R Fangerau H Seitz RJ: “Broca’s area” as a collective term? *Brain Lang.* 2007;102(1):22–29. 10.1016/j.bandl.2006.11.012 17257665

[ref-71] Lindenberg R Uhlig M Scherfeld D: Communication with emblematic gestures: shared and distinct neural correlates of expression and reception. *Hum Brain Mapp.* 2012;33(4):812–823. 10.1002/hbm.21258 21484956 PMC6870381

[ref-72] Litt A Plassmann H Shiv B: Dissociating valuation and saliency signals during decision-making. *Cereb Cortex.* 2011;21(1):95–102. 10.1093/cercor/bhq065 20444840

[ref-73] Mériau K Wartenburger I Kazzer P: A neural network reflecting individual differences in cognitive processing of emotions during perceptual decision making. *Neuroimage.* 2006;33(3):1016–1027. 10.1016/j.neuroimage.2006.07.031 16973382

[ref-74] Milham MP Banich MT Barad V: Competition for priority in processing increases prefrontal cortex’s involvement in top-down control: an event-related fMRI study of the stroop task. *Brain Res Cogn Brain Res.* 2003;17(2):212–222. 10.1016/S0926-6410(03)00108-3 12880892

[ref-75] Miller EK Cohen JD: An integrative theory of prefrontal cortex function. *Annu Rev Neurosci.* 2001;24:167–202. 10.1146/annurev.neuro.24.1.167 11283309

[ref-76] Morewedge CK Kahneman D: Associative processes in intuitive judgment. *Trends Cogn Sci.* 2010;14(10):435–440. 10.1016/j.tics.2010.07.004 20696611 PMC5378157

[ref-77] Moskowitz GB Stone J Childs A: Implicit stereotyping and medical decisions: unconscious stereotype activation in practitioners’ thoughts about African Americans. *Am J Public Health.* 2012;10(5):996–1001. 10.2105/AJPH.2011.300591 22420815 PMC3325336

[ref-78] Murphy ST Zajonc RB: Affect, cognition, and awareness: affective priming with optimal and suboptimal stimulus exposures. *J Pers Soc Psychol.* 1993;64(5):723–739. 10.1037/0022-3514.64.5.723 8505704

[ref-79] Nomura M Ohira H Haneda K: Functional association of the amygdala and ventral prefrontal cortex during cognitive evaluation of facial expressions primed by masked angry faces: an event-related fMRI study. *Neuroimage.* 2004;21(1):352–363. 10.1016/j.neuroimage.2003.09.021 14741673

[ref-80] Oldfield RC: The assessment and analysis of handedness: the Edinburgh inventory. *Neuropsychologia.* 1971;9(1):97–113. 10.1016/0028-3932(71)90067-4 5146491

[ref-81] Opialla S Lutz J Scherpiet S: Neural circuits of emotion regulation: a comparison of mindfulness-based and cognitive reappraisal strategies. *Eur Arch Psychiatry Clin Neurosci.* 2015;265(1):45–55. 10.1007/s00406-014-0510-z 24902936

[ref-82] Opitz B Mecklinger A Friederici AD: Functional asymmetry of human prefrontal cortex: encoding and retrieval of verbally and nonverbally coded information. *Learn Mem.* 2000;7(2):85–96. 10.1101/lm.7.2.85 10753975 PMC311325

[ref-83] Plassmann H O’Doherty J Rangel A: Orbitofrontal cortex encodes willingness to pay in everyday economic transactions. *J Neurosci.* 2007;27(37):9984–9988. 10.1523/JNEUROSCI.2131-07.2007 17855612 PMC6672655

[ref-84] Prochnow D Brunheim S Kossack H: Statistical data of subareas of the dorsolateral frontal cortex in socially relevant decisions based on masked affect expressions. *figshare.* 2014a Data Source 10.12688/f1000research.4734.1 PMC4516020 26236464

[ref-85] Prochnow D Höing B Kleiser R: The neural correlates of affect reading: an fMRI study on faces and gestures. *Behav Brain Res.* 2013a;237:270–277. 10.1016/j.bbr.2012.08.050 22981562

[ref-86] Prochnow D Kossack H Brunheim S: Processing of subliminal facial expressions of emotion: a behavioral and fMRI study. *Soc Neurosci.* 2013b;8(5):448–461. 10.1080/17470919.2013.812536 23869578

[ref-87] Prochnow D Steinhäuser L Brunheim S: Differential emotional state reasoning in young and older adults: Evidence from behavioral and neuroimaging data. *J Neurol Psychol.* 2014b;2(1):1–8. Reference Source

[ref-88] Reniers RL Corcoran R Völlm BA: Moral decision-making, ToM, empathy and the default mode network. *Biol Psychol.* 2012;90(3):202–210. 10.1016/j.biopsycho.2012.03.009 22459338

[ref-89] Rizzolatti G Craighero L: The mirror-neuron system. *Annu Rev Neurosci.* 2004;27:169–192. 10.1146/annurev.neuro.27.070203.144230 15217330

[ref-90] Rohr M Degner J Wentura D: Masked emotional priming beyond global valence activations. *Cogn Emot.* 2012;26(2):224–244. 10.1080/02699931.2011.576852 21970398

[ref-91] Rottschy C Langner R Dogan I: Modelling neural correlates of working memory: a coordinate-based meta-analysis. *Neuroimage.* 2012;60(1):830–846. 10.1016/j.neuroimage.2011.11.050 22178808 PMC3288533

[ref-92] Ruys KI Aarts H: I didn’t mean to hurt you! Unconscious origins of experienced self-agency over others’ emotions. *Emotion.* 2012;12(1):132–141. 10.1037/a0023161 21707153

[ref-93] Sanfey AG: Social decision-making: insights from game theory and neuroscience. *Science.* 2007;318(5850):598–602. 10.1126/science.1142996 17962552

[ref-94] Schulte-Rüther M Markowitsch HJ Fink GR: Mirror neuron and theory of mind mechanisms involved in face-to-face interactions: a functional magnetic resonance imaging approach to empathy. *J Cogn Neurosci.* 2007;19(8):1354–1372. 10.1162/jocn.2007.19.8.1354 17651008

[ref-95] Seitz RJ Franz M Azari NP: Value judgments and self-control of action: the role of the medial frontal cortex. *Brain Res Rev.* 2009;60(2):368–378. 10.1016/j.brainresrev.2009.02.003 19285106

[ref-96] Seitz RJ Nickel J Azari NP: Functional modularity of the medial prefrontal cortex: involvement in human empathy. *Neuropsychology.* 2006;20(6):743–751. 10.1037/0894-4105.20.6.743 17100519

[ref-97] Seitz RJ Schäfer R Scherfeld D: Valuating other people’s emotional face expression: a combined functional magnetic resonance imaging and electroencephalography study. *Neuroscience.* 2008;152(3):713–722. 10.1016/j.neuroscience.2007.10.066 18313858

[ref-98] Shamay-Tsoory SG Aharon-Peretz J Perry D: Two systems for empathy: a double dissociation between emotional and cognitive empathy in inferior frontal gyrus versus ventromedial prefrontal lesions. *Brain.* 2009;132(Pt 3):617–627. 10.1093/brain/awn279 18971202

[ref-99] Shen XB Wu Q Fu XL: Effects of the duration of expressions on the recognition of microexpressions. *J Zhejiang Univ Sci B.* 2012;13(3):221–230. 10.1631/jzus.B1100063 22374615 PMC3296074

[ref-100] Shibasaki H Hallett M: What is the Bereitschaftspotential? *Clin Neurophysiol.* 2006;117(11):2341–2356. 10.1016/j.clinph.2006.04.025 16876476

[ref-101] Silvers JA Weber J Wager TD: Bad and worse: neural systems underlying reappraisal of high- and low-intensity negative emotions. *Soc Cogn Affect Neurosci.* 2015;10(2):172–9. 10.1093/scan/nsu043 24603024 PMC4321618

[ref-102] Slotnick SD White RC: The fusiform face area responds equivalently to faces and abstract shapes in the left and central visual fields. *Neuroimage.* 2013;83:408–417. 10.1016/j.neuroimage.2013.06.032 23777758

[ref-103] Smith DV Hayden BY Truong TK: Distinct value signals in anterior and posterior ventromedial prefrontal cortex. *J Neurosci.* 2010;30(7):2490–2495. 10.1523/JNEUROSCI.3319-09.2010 20164333 PMC2856318

[ref-104] Sokol-Hessner P Hutcherson C Hare T: Decision value computation in DLPFC and VMPFC adjusts to the available decision time. *Eur J Neurosci.* 2012;35(7):1065–1074. 10.1111/j.1460-9568.2012.08076.x 22487036 PMC3325500

[ref-105] Suslow T Kugel H Ohrmann P: Neural correlates of affective priming effects based on masked facial emotion: an fMRI study. *Psychiatry Res.* 2013;211(3):239–245. 10.1016/j.pscychresns.2012.09.008 23131525

[ref-106] Svenson O: Decision Making and the Search for Fundamental Psychological Regularities: What Can Be Learned from a Process Perspective? *Organ Behav Hum Decis Process.* 1996;65(3):252–267. 10.1006/obhd.1996.0026

[ref-107] Sweeny TD Grabowecky M Suzuki S: Long-lasting effects of subliminal affective priming from facial expressions. *Conscious Cogn.* 2009;18(4):929–938. 10.1016/j.concog.2009.07.011 19695907 PMC2784103

[ref-108] Talairach J Tournoux P: Co-planar stereotaxic atlas of the human brain. Stuttgart: Thieme.1988 Reference Source

[ref-109] Thirioux B Mercier MR Blanke O: The cognitive and neural time course of empathy and sympathy: an electrical neuroimaging study on self-other interaction. *Neuroscience.* 2014;267:286–306. 10.1016/j.neuroscience.2014.02.024 24583040

[ref-110] Turkeltaub PE Eickhoff SB Laird AR: Minimizing within-experiment and within-group effects in Activation Likelihood Estimation meta-analyses. *Hum Brain Mapp.* 2012;33(1):1–13. 10.1002/hbm.21186 21305667 PMC4791073

[ref-111] van’t Wout M Kahn RS Sanfey AG: Repetitive transcranial magnetic stimulation over the right dorsolateral prefrontal cortex affects strategic decision-making. *Neuroreport.* 2005;16(16):1849–1852. 10.1097/01.wnr.0000183907.08149.14 16237340

[ref-112] Wagner AD Maril A Bjork RA: Prefrontal contributions to executive control: fMRI evidence for functional distinctions within lateral Prefrontal cortex. *Neuroimage.* 2001;14(6):1337–1347. 10.1006/nimg.2001.0936 11707089

[ref-113] Walter H Adenzato M Ciaramidaro A: Understanding intentions in social interaction: the role of the anterior paracingulate cortex. *J Cogn Neurosci.* 2004;16(10):1854–1863. 10.1162/0898929042947838 15701234

[ref-114] Watson D Clark LA Tellegren A: Development and validation of brief measures of positive and negative affect: the PANAS scales. *J Pers Soc Psychol.* 1988;54(6):1063–1070. 10.1037/0022-3514.54.6.1063 3397865

[ref-115] Winkielman P Berridge KC Wilbarger JL: Unconscious affective reactions to masked happy versus angry faces influence consumption behavior and judgments of value. *Pers Soc Psychol Bull.* 2005;31(1):121–135. 10.1177/0146167204271309 15574667

[ref-116] Worsley KJ Evans AC Marrett S: A three-dimensional statistical analysis for CBF activation studies in human brain. *J Cereb Blood Flow Metab.* 1992;12(6):900–918. 10.1038/jcbfm.1992.127 1400644

[ref-117] Wunderlich K Rangel A O’Doherty JP: Economic choices can be made using only stimulus values. *Proc Natl Acad Sci U S A.* 2010;107(34):15005–15010. 10.1073/pnas.1002258107 20696924 PMC2930519

[ref-118] Xi C Zhu Y Niu C: Contributions of subregions of the prefrontal cortex to the theory of mind and decision making. *Behav Brain Res.* 2011;221(2):587–593. 10.1016/j.bbr.2010.09.031 20934455

[ref-119] Zelazo PD Muller U: Executive function in typical and atypical development. In Goswami U. *Handbook of childhood cognitive development* Oxford: Blackwell.2002;445–489. 10.1002/9780470996652.ch20

[ref-120] Zhu Q Nelissen K Van den Stock J: Dissimilar processing of emotional facial expressions in human and monkey temporal cortex. *Neuroimage.* 2013;66:402–411. 10.1016/j.neuroimage.2012.10.083 23142071 PMC3625447

