# Supplementary material for: Anterior and posterior subareas of the dorsolateral frontal cortex in socially relevant decisions based on masked affect expressions
Source: F1000Res. 2015 Jul 9;3:212. Originally published 2014 Sep 5. [Version 3] doi: 10.12688/f1000research.4734.3 (PMC4516020; doi:10.12688/f1000research.4734.3)
Supplement: Peer review history

# Peer review: Referee response for version 3

**DOI:** 10.5256/f1000research.7214.r9430

**Published:** 2015-07-27

**Authors:** Gonzalo G de Polavieja

**Affiliations:** 1 Neuroscience Programme, Champalimaud Foundation, Lisbon, Portugal

I am happy with the addition in the Limitations section and with a better description of the controls. I still think in future studies other controls can be added with procedures that still leave face-like features but eliminate affect expressions.

I have read this submission. I believe that I have an appropriate level of expertise to confirm that it is of an acceptable scientific standard.

# Peer review: Referee response for version 3

**DOI:** 10.5256/f1000research.7214.r9457

**Published:** 2015-07-13

**Authors:** Motoaki Sugiura

**Affiliations:** 1 Institute of Development, Aging and Cancer, Tohoku University, Sendai, Japan

I have read this submission. I believe that I have an appropriate level of expertise to confirm that it is of an acceptable scientific standard.

# Peer review: Referee response for version 2

**DOI:** 10.5256/f1000research.6841.r8390

**Published:** 2015-05-14

**Authors:** Gonzalo G de Polavieja

**Affiliations:** 1 Neuroscience Programme, Champalimaud Foundation, Lisbon, Portugal

I thank the authors for careful consideration of my previous comments. However, I still think that my main concern remains. The comparison of the reaction to faces against scrambled images is not enough to conclude that the difference in activation is due to faces. It could well be due to any characteristic in the 'faces' class not present in the 'scrambled' class, like smoothness or local contrast values. They mention in their response that scrambled images are are 'suited to maximize the cognitive comparison to faces'. Quite the opposite, you would need a control class that has as much as possible from the 'faces' class except the appearance of faces, probably several classes of distorted faces as well as your present control.

I have read this submission. I believe that I have an appropriate level of expertise to confirm that it is of an acceptable scientific standard, however I have significant reservations, as outlined above.

# Peer review: Referee response for version 2

**DOI:** 10.5256/f1000research.6841.r8483

**Published:** 2015-04-28

**Authors:** Motoaki Sugiura

**Affiliations:** 1 Institute of Development, Aging and Cancer, Tohoku University, Sendai, Japan

This reviewer is happy with the authors’ effort on the revision and the resulting significant improvement of the manuscript. However, several important issues are remained unanswered, as follow:

For point 1, the authors clarified that the two contrasts do represent the cognitive process of their interest (i.e., two components of decision making process) but failed to provide reasonable defense against the contamination of the process of no interest, which was the point of this reviewer’s criticism; for example, as the reviewer 1 has also pointed out, the contrast against the scrambled face includes many decision-irrelevant processes including object or face perception. The authors have to explain the reason to believe that the two contrasts predominantly reflect the process of their interest rather than other confounding factors, and also describe the unfavorable possibility as a limitation in the Discussion section.

For point 3, the reasons for the difference between the case 1 and case 2 are understandable, but not for the case 3. Please provide the reason for the necessity of Monte-Carlo-like simulation. In addition, please provide these reasons also for readers explicitly in the manuscript.

For point 4, considering the slow hemodynamic-response “temporal separation of 2.600 ms on average” is obviously insufficient to make two time-series models independent, regardless of the scanning TR. Here the detailed information on the range of jittering is important, and this limitation caused by this insufficient separation should be declared in the Discussion section

I have read this submission. I believe that I have an appropriate level of expertise to confirm that it is of an acceptable scientific standard, however I have significant reservations, as outlined above.

# Peer review: Referee response for version 1

**DOI:** 10.5256/f1000research.5056.r7367

**Published:** 2015-03-03

**Authors:** Gonzalo G de Polavieja

**Affiliations:** 1 Neuroscience Programme, Champalimaud Foundation, Lisbon, Portugal

The authors use fMRI experiments to argue that the dorsolateral frontal cortex (DLFC) responds in decision-making to masked affect human faces.

1. The reference images for the pre-decision phase are scrambled images. Scrambled images have a number of obvious features as they are not (a), faces, (b) smooth and (c) they have local contrast different to faces. So it is not obvious to me why the experimental condition, respect to this baseline, corresponds to affect faces, and not more generally to (a) some structure of faces, (b) smooth figures or (c) particular contrast structures. Why wouldn't you need to use additional controls to eliminate these options? (i.e. normal faces or objects)
  Related to this, you mention that you eliminate the 26% of subjects that suspect the presence of emotional faces and that 78% reported flickering. Although I didn't understand whether this flickering came from real flickering or not, I could not find in the text whether some subjects could report seeing the transition from scramble image to face. 
2. Some of the reported data (mean age, % subjects that suspect emotional faces, etc) are identical to Prochnow e *t al *2013b, but I cannot find mention in the present ms that the data collected is the same than in Prochnow *et al* 2013b or that the subjects are the same. Is this the case? Please, clarify.
3. Data presented are comparison of means, but the data obtained is distributions. Did you find whether other parameters of the distributions for other brain regions were significant (median, mode, variance).
4. Is there individual variability? As you made many preliminary tests on subjects (for example, empathy), it would be very interesting to correlate these results with brain activations.

I have read this submission. I believe that I have an appropriate level of expertise to confirm that it is of an acceptable scientific standard, however I have significant reservations, as outlined above.

# Peer review: Referee response for version 1

**DOI:** 10.5256/f1000research.5056.r6218

**Published:** 2014-09-25

**Authors:** Motoaki Sugiura

**Affiliations:** 1 Institute of Development, Aging and Cancer, Tohoku University, Sendai, Japan

The authors tried to demonstrate that anterior and posterior subregions of the DLFC are involved in different phases (i.e., pre-decision and decision *per se*, respectively) of socially relevant decisions based on subliminal emotional information. The addressed issue is important in related fields and the approach is unique. However, I consider that the following methodological issues require clarification before the evaluation of the work.

Major points:

1. Is it reasonable to use the contrasts “masked facial expressions vs. baseline” and “decisions based on masked affect expressions vs. motor control” to isolate the DLPF subregions for the socially relevant decisions based on subliminal emotional information? Activation identified in these contrasts may be affected by variety of perceptual and cognitive processes. How can the authors’ assumption that activation in these contrasts reflected only the decision making process be justified?
2. Also, in the MACM approach, authors did not make the selection of the data based on the task or contrast. How, then, can the authors’ argue the identified connectivity specifically reflected the decision making process?
3. The authors used 1) “p < 0.005 and minimal cluster size of 405 voxels” for the fMRI analysis, 2) “a family-wise error (FWE) corrected p-threshold of p < 0.05” for the MACM/ALE analysis, and 3) some Monte-Crlo-like simulation described after the explanation of 2. I don’t understand the reason for using both 2 and 3 for the MACM/ALE analysis, and also see how adaptation of these three different thresholds/criteria across three cases was justified.
4. In the task, how can activation for the pre-decision and decision phases be separated despite the fixed close interval of the two phases?
5. Region of interest (ROI) analysis, paragraph 2 - “We conducted pairwise t-tests to…”: Why was this uncommon analysis necessary?

Details:

- Introduction, paragraph 3, last sentence - “Recent evidence, however, suggests that gaming and ToM scenarios are based at least partly on different neural circuits”: I don’t get the nuance; was there an assumption that the gaming and ToM perfectly share the neural substrate?
- FMRT data analysis, paragraph 3 - “To ensure comparability…”: I don’t understand what is meant by “comparability” (i.e., with what?) or what has exactly been done here. In addition, authors should be clear about the “template” for the normalization; “Talairach space” sometimes means just a 3D brain space.
- Functional connectivity analyses, paragraph 1 - “above-mentioned criteria”: which criteria do you mean?

I have read this submission. I believe that I have an appropriate level of expertise to confirm that it is of an acceptable scientific standard, however I have significant reservations, as outlined above.
